# Applying, Evaluating and Refining Bioinformatics Core Competencies (An Update from the Curriculum Task Force of ISCB’s Education Committee)

**DOI:** 10.1371/journal.pcbi.1004943

**Published:** 2016-05-13

**Authors:** Lonnie Welch, Cath Brooksbank, Russell Schwartz, Sarah L. Morgan, Bruno Gaeta, Alastair M. Kilpatrick, Daniel Mietchen, Benjamin L. Moore, Nicola Mulder, Mark Pauley, William Pearson, Predrag Radivojac, Naomi Rosenberg, Anne Rosenwald, Gabriella Rustici, Tandy Warnow

**Affiliations:** 1 School of Electrical Engineering and Computer Science, Ohio University, Athens, Ohio, United States of America; 2 European Molecular Biology Laboratory, European Bioinformatics Institute, Wellcome Genome Campus, Hinxton, Cambridge, United Kingdom; 3 Department of Biological Sciences and Computational Biology Department, Carnegie Mellon University, Pittsburgh, Pennsylvania, United States of America; 4 School of Computer Science and Engineering, UNSW, Sydney, New South Wales, Australia; 5 Department of Pediatrics, University of California San Diego, La Jolla, California, United States of America; 6 National Institutes of Health, Bethesda, Maryland, United States of America; 7 MRC Institute of Genetics and Molecular Medicine, University of Edinburgh, Edinburgh, United Kingdom; 8 Computational Biology group, Department of Integrative Biomedical Sciences, IDM, University of Cape Town, Cape Town, South Africa; 9 School of Interdisciplinary Informatics, University of Nebraska at Omaha, Omaha, Nebraska, United States of America; 10 Department of Biochemistry and Molecular Genetics, School of Medicine, University of Virginia, Charlottesville, Virginia, United States of America; 11 School of Informatics and Computing, Indiana University, Bloomington, Indiana, United States of America; 12 Tufts University School of Medicine, Boston, Massachusetts, United States of America; 13 Department of Biology, Georgetown University, Washington, D.C., United States of America; 14 School of the Biological Sciences, University of Cambridge, Cambridge Systems Biology Centre, Cambridge, United Kingdom; 15 Departments of Computer Science and Bioengineering, University of Illinois, Urbana, Illinois, United States of America

The Curriculum Task Force (CTF) of ISCB’s Education Committee seeks to define curricular guidelines for those who educate or train bioinformatics professionals at all career stages. A recent report of the CTF [1] presented a draft set of bioinformatics core competencies, derived from the results of surveys of (1) core facility directors, (2) career opportunities, and (3) existing curricula.

Since the publication of its 2014 report, the CTF has focused on the application of the guidelines in varied contexts to identify areas where refinement is needed. As a first step, the task force held an open meeting at the ISMB conference in July 2014. The ideas discussed at the meeting spawned four working groups (WGs), which focus on (i) defining core competencies for specific types and levels of bioinformatics training, (ii) mapping the curriculum guidelines and competencies to existing materials in order to identify the need for development of new materials, and (iii) identifying where revision of the guidelines may be valuable. The CTF is engaging the ISCB community through open WG meetings at ISCB’s official conferences. Thus far, the WGs have convened at the ISCB Great Lakes Bioinformatics Conference (Purdue University, May 2015) and at the ISMB/ECCB Conference (Dublin, Ireland, July 2015). Additionally, the CTF held a workshop at the Annual General Meeting of the Global Organization of Bioinformatics Learning, Education and Training (Cape Town, South Africa, November 2015). Specifically, the draft competencies have been employed in a wide range of activities and contexts (see Table 1 and [2–11]), including the development of new curricula, the analysis of existing curricula, and the creation of new roles involving bioinformatics. These activities have resulted in the identification of several areas where refinement would be useful:

- ***Identify different levels or phases of competency***. It would be helpful to define different phases of competency development, or different levels of competency appropriate for distinct roles.
- ***Define competency profiles for disciplines that don’t fit into our current silos***. Bioengineering provides an illustrative example of a discipline that requires core competency in bioinformatics but does not fit into our current categories. There are almost certainly others. It would be helpful if we could provide some guidance on how to produce ‘hybrid’ competency profiles, perhaps borrowing some competencies from the TF’s core set and others from different disciplines. The LifeTrain initiative (www.lifetrain.eu) [2, 3] is collecting competency profiles for a range of disciplines of relevance to the biomedical sciences and may provide a useful resource kit for this.
- ***Broaden the scope of the competency profiles in response to cutting-edge and emerging research***. Current areas requiring improvement include incorporating competencies that capture a fundamental understanding of the biological principles central to analyzing biomolecular data, and broadening the user WG to include applications beyond medicine.
- ***Provide guidance on the evidence required to assess whether someone has acquired each competency***. For undergraduate, Master’s and PhD programs, learning outcomes for each competency, perhaps with examples of appropriate means of assessment, would be valuable. For established professionals who need to assimilate competencies into their working lives, a different approach may be required (such as keeping a portfolio to capture evidence of competency); the CTF should seek guidance from relevant professional bodies, especially in regulated professions such as healthcare.
- ***Provide indicative course content or examples of programs that map to the competency requirements***. We do not wish to prescribe what course providers should teach or how they should teach it; however, if a course provider is designing a course to meet a specific competency requirement, it may be helpful to find examples of other programs that do this successfully. One way of achieving this is by mapping existing training content to the TF’s competencies. Another way might be to provide an indication, perhaps based on several courses, of the course content that would meet the competency requirements. This would give course providers the freedom to build their own course syllabi without having to reinvent the wheel. Initiatives to collect examples of Creative Commons (or otherwise reusable) course materials will provide an extremely valuable bank of training materials that could be mapped to the core competencies.

**Table 1:** Summary of the activities of the ISCB Curriculum Task Force.

Organization	Program (level-U/G/P)	Activities	Working Group(s)
American Association of Medical Colleges	Graduate Research Education and Training Group-G	• bioinformatics education for academic medicine	User (physician-scientist)
		• assessment	
		• levels of competency	
Carnegie Mellon University	Biology-U	• core computational biology class (biologists)	Scientist
		• computation throughout biology curricula	
Carnegie Mellon/University of Pittsburgh	PhD Program in Computational Biology-G	• a model for the expectations of entering students	Engineer
EMBL-EBI	Professional courses-P	• mapping courses to competencies	Content
GOBLET	Learning, Educ & Training-P	• training portal for course information and materials	Content
H3ABioNet	Pan African Bioinformatics network-G	• identifying modules	Engineer, Scientist, User
		• foundation for developing content	
Health Education England	England’s clinical bioinformatics working group-P	• defining the role of a clinical bioinformatician	User (healthcare professional)
		• enabling use of bioinformatics for clinical decision-making	
Indiana University	Bioinformatics Programs-G	• making discipline-centric courses interdisciplinary	Engineer, Scientist
Network for Bioinformatics in Life Sci. Education	NSF-funded network of investigators-U	• integrating bioinformatics into life sciences curricula	Scientist
Ohio University	bioinformatics certificates-U/G	• training for each bioinformatics role	Engineer, Scientist, User
Springer	ISCB book series	• mapping	Content
		• identifying needs	
University of Cambridge		• mapping courses to competencies	Content, Scientist
		• integrating bioinformatics into life sciences curricula	
University of Illinois	Bioengineering-U	• training bioengineers	Engineer
University of New South Wales	Bioinformatics Engineering-U	• program design and accreditation	Engineer
University of Virginia	Bioinformatics Course -G	• biological principles for analyzing genomic data	Scientist
WikiProject Computational Biology	Wikipedia, Wikidata, and other Wikimedia projects	• organizing articles	Content
		• crowd curation	
		• mapping	
		• strategic planning	
